# Interplay Between Dietary Phytochemicals and Gut Microbiota in Colorectal Cancer: Future Directions and Considerations

**DOI:** 10.1155/jnme/5512453

**Published:** 2025-10-28

**Authors:** Fernando Tume, Edgardo J. Palma-Gutierrez, Román Acevedo-Espinola, Marysol Olivares-Etchebaster

**Affiliations:** ^1^Research Group in Metabolism and Molecular Nutrition, Universidad Científica del Sur, Lima, Peru; ^2^Department of Nutrition and Dietetics, Universidad Científica del Sur, Lima, Peru

**Keywords:** colorectal neoplasms, metabolites, microbiota, phytochemicals

## Abstract

Colorectal cancer (CRC) is one of the most lethal cancers worldwide. Its development and progression are influenced by a combination of genetic and environmental factors such as diet. Recent studies highlight the role of dietary phytochemicals in the prevention and treatment of CRC. In this review, we explore the role of phytochemicals in directly targeting CRC and examine how microbiota convert these phytochemicals into bioactive metabolites. These metabolites may play a preventive and therapeutic role against CRC through cellular, molecular, and epigenetic mechanisms. We emphasize the need for additional actions to translate the existing knowledge effectively and safely into complementary treatments for CRC patients. The incorporation of phytochemical-rich diets represents a viable option in the battle against this debilitating condition.

## 1. Introduction

Colorectal cancer (CRC) is one of the most prevalent neoplasms worldwide, responsible for 909,019 deaths in 2022. According to the World Health Organization, it ranks as the second leading cause of cancer-related mortality [1]. In the United States alone, an estimated 153,020 new cases and 52,550 deaths were projected for 2023 [2]. Notably, 12.8% of these new diagnoses and 7.1% of the deaths were estimated to belong to individuals under the age of 50, highlighting a concerning trend in younger populations [3]. Given the inherent side effects of conventional treatment options like chemotherapy, the demand for safe, effective, and cost-efficient alternatives has become increasingly urgent [4]. So, the use of such alternatives in a complementary and synergistic manner is strongly advocated [5].

Although genetic susceptibility plays a significant role in the development and progression of this malignancy, environmental factors, particularly diet, also significantly contribute throughout the lifespan of the patient [6–9]. In this regard, some dietary patterns, such as the Mediterranean diet, exhibit notable benefits in reducing the risk of CRC onset [10]. This diet includes a variety of healthy, plant-derived foods, rich in bioactive compounds that modulate cellular and molecular mechanisms associated with cancer cell proliferation, invasion, metastasis, and immune evasion [11]. In this context, phytochemicals are one of the most bioactive substances that, despite not being considered nutrients, play an important role in maintaining cellular homeostasis [12] and have been tested in numerous pathologies, including CRC [13, 14].

**Figure 1:**
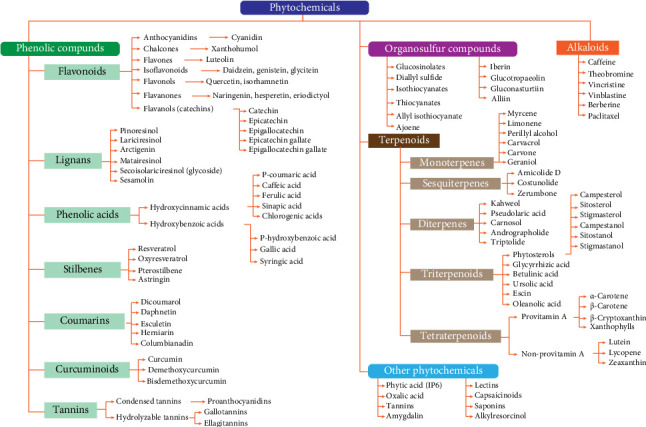
Classification of the main dietary phytochemicals.

In a broader definition, phytochemicals represent a class of biologically active compounds inherent to plants produced by their primary or secondary metabolism [15]. This diverse assemblage of compounds contributes to the vibrant color, distinct taste, and characteristic aroma observed in numerous fruits, vegetables, and herbs. Furthermore, they serve as crucial elements in plant defense mechanisms against external pathogens, including microorganisms [12]. The primary groups of phytochemicals extensively documented in epidemiological, *in vivo*, and *in vitro* investigations comprise members of phenolic compounds (e.g., flavonoids, phenolic acids, curcumin, lignans, and stilbenes), terpenoids (e.g., carotenoids, limonene, and phytosterols), organosulfur compounds (OSCs), and alkaloids (Figure 1) [15–17].

Certain phytochemicals target biochemical pathways dysregulated in malignancies such as CRC [18, 19]. Additionally, due to their low solubility and bioavailability in the small intestine, these compounds may reach the colon, where they act as direct modulators of gut microbiota and/or are metabolized by microbial enzymes into substances that could potentially influence CRC-related hallmarks. It is important to acknowledge that the retention time and the effects of these phytochemicals depend on individual differences in metabotype, which are shaped by gut microbiota composition and dietary habits [18, 20]. Clinical trials rarely stratify participants by metabotype, despite evidence that microbial capacity to generate metabolites such as equol dictates interindividual responses [20]. Moreover, most studies focus on fecal readouts rather than colonic tissue concentrations or mucosa-associated microbiota, limiting mechanistic insight [21].

In this paper, we reviewed the interplay between microbiota, phytochemicals, and CRC. First, we discuss the anti-CRC properties of the phytochemicals that are naturally present in foods, as these are the most relevant to human health. Second, we approach how phytochemicals are metabolized into potential bioactive compounds that target CRC. Finally, by bridging mechanistic insights with nutritional and clinical perspectives, this review outlines some considerations for a potential safe and efficient use of phytochemicals as part of nutritional regimes or supplementation in healthy individuals and CRC patients.

## 2. CRC and Phytochemicals

Cancer has special features known as the hallmarks of cancer. They are a set of cellular and molecular characteristics that normal cells acquire as they progressively evolve toward a malignant state [22–24]. In CRC, these features play a role in the initiation, promotion, progression, and metastasis [6]. Preclinical evidence and clinical evidence point out that healthy dietary components are capable of directly counteracting some of the cancer features, such as proliferative signaling, immune evasion, tumor-promoting inflammation, and complex microbial ecosystems [6]. In this context, the use of phytochemicals for the prevention or treatment of cancer is part of the “chemopreventive” strategy that aims at reducing the incidence and development of cancer [25–27]. Fruits and vegetables are excellent sources of phytochemicals, and their consumption is associated with a lower risk of CRC [28–30]. A systematic review that included more than one million participants showed that high consumption of fruits such as kiwi, citrus, watermelon, and apples is inversely correlated with CRC risk [31]. Moreover, a study by Alzate-Yepes et al. [32] highlights the molecular mechanisms of phytochemicals found in fruits and vegetables, supporting their consumption as a rational chemopreventive strategy against CRC.

### 2.1. Phenolic Compounds

Most compounds in this family have more than two phenolic units and are therefore referred to as polyphenols. Studies found that dietary phenolic compounds, particularly flavonoids, are associated with a reduced risk of CRC [33]. Examples of widely studied phenolic compounds are resveratrol [34], curcumin [35], sinapic acid [13], ellagitannins [36, 37], procyanidin C1 [38], and epigallocatechin gallate (EGCG) [33]. They are considered potential chemopreventive agents against CRC due to their anti-inflammatory, antioxidant, antiproliferative, and proapoptotic activities [4, 13, 14, 33]. *In vitro* and *in vivo* studies show that these plant-derived compounds can modulate multiple biochemical pathways involved in CRC development, including Wnt/β-catenin, MAPK, PI3K/Akt, Akt/mTOR, EGFR/STAT/ERK, and NF-κB pathways [4, 18, 39–41]. Luo et al. [42] reported that EGCG, the primary active polyphenol in green tea, inhibited proliferation of CRC cell lines by inducing apoptosis in a dose-dependent manner and also suppressed migration *in vitro*. In that same study, they further showed that EGCG treatment led to downregulation of STAT3 and phosphorylated STAT3, along with reductions in antiapoptotic proteins such as Bcl-2 and MCL-1, and the mesenchymal marker vimentin, while increasing E-cadherin. In the clinical setting, green tea extract containing EGCG demonstrated a favorable safety profile but did not exhibit statistically significant differences in decreasing the colorectal adenoma rate among German participants after 3 years of follow-up [43]. Similarly, a phase II clinical trial conducted in the United States indicates that a preparation containing EGCG was ineffective in preventing rectal aberrant crypt foci [44]. These unsatisfactory clinical results raise important questions about the factors that should be considered when translating preclinical findings to the clinical arena.

Ellagitannins are a type of tannin found in various edible food sources. Pajari et al. [45] tested ellagitannin-rich cloudberry (*Rubus chamaemorus*) extract in an animal model of intestinal neoplasia. After 10 weeks of cloudberry feeding, they observed a reduction in tumor growth. Notably, this antitumorigenic effect appeared to be mediated, at least partially, by the downregulation of the hepatocyte growth factor signaling pathway, suggesting a potential role for ellagitannins in interrupting the transition toward a metastatic phenotype in colon cancer cells. While the authors acknowledge the possibility of synergistic interactions with other phytochemicals present in the extract, the current findings paved the way for further research into the potential anti-CRC properties of ellagitannin-rich fruits beyond cloudberry. Indeed, in a randomized controlled trial, Nuñez-Sánchez et al. [46] demonstrated that ellagitannin-containing pomegranate extract supplementation altered the expression of cancer-related genes (e.g., *CD44*, *CTNNB1*, and *EGFR*) in colon tissues from CRC patients. The authors acknowledged limitations to the study including a small sample size (*n* = 35 patients, *n* = 10 controls) and substantial interindividual variability, highlighting the need for larger and more controlled studies to confirm these findings.

In the onset, progression, and metastasis of CRC, epigenetics plays a fundamental role [47, 48]. Epigenetics refers to the regulation of gene expression without changing the nucleotide sequencing. These mechanisms include methylation/demethylation of DNA and histones and acetylation/deacetylation of histones and noncoding RNAs such as microRNA and long noncoding RNA (lncRNA) [49, 50]. Phenolic compounds target epigenetics mechanisms disrupted in CRC. Genistein, an isoflavone found in legumes, has shown preventive benefits against colon dysfunction, mechanistically through modulating immune function, mucus production, and cell proliferation [51], and experimental studies highlight the importance of this phytochemical as an epigenetic regulator in CRC development [52]. More specifically, a study by Zhu et al. [53] found that genistein epigenetically rescues the function of the tumor suppressor gene *WIF1* to prevent the invasion and migration of malignant colon cells. Other phenolic compounds such as ellagic acid have shown anti-CRC properties by regulating lncRNAs related to apoptosis [54].

Furthermore, depending on the disease stage, drug resistance often develops in CRC following conventional treatments. Dietary phenolic compounds could sensitize CRC cells to chemotherapeutic agents and enhance treatment efficacy [5]. For example, resveratrol sensitizes CRC cells to 5-fluorouracil (5-FU) by targeting the biochemical pathway of the angiogenic factor HIF-1α [55]. Moreover, curcumin and calebin A from turmeric (*Curcuma longa*) have also been reported as chemosensitizers [56]. These findings highlight another advantage of using phytochemical-rich diets as a complement instead of only using first-line chemotherapeutics [55].

### 2.2. OSCs

Glucosinolates, known for their multiple anticancer properties, are key compounds in this class. By preparation, chewing, cooking, or digestion of vegetables, they are transformed into active biological metabolites such as thiocyanates, isothiocyanates (ITCs), and nitriles that exhibit inhibitory properties against CRC [57]. *In vitro* experiments demonstrated that ITCs-rich extracts from cauliflower and radish significantly inhibited the metabolic activity of CRC cell lines, with minimal inhibitory effects observed on noncancerous colon epithelial cells [58]. Building upon the well-known anti-CRC activities of ICTs, Kim et al. [59] generated *Brassica rapa* lines through traditional breeding, resulting in phenotypes with high levels of ITCs. Extracts obtained from these lines exhibited potent antiproliferative activity against colon cancer cell lines and further analysis revealed downregulation of the NF-κB signaling pathway. Consistent with this experimental evidence, a prospective study conducted among Chinese men found that elevated levels of urinary ITCs are associated with a reduced risk of CRC, thereby raising questions about the metabolism of these bioactive molecules [60].

Garlic is a major dietary source of OSCs such as alliin, allicin, S-allylcysteine, and various allyl sulfides. These phytochemicals are released or transformed during processing and digestion, and in experimental models, they have been shown to counteract cancer hallmarks including mutagenesis, oxidative stress, chronic inflammation, and aberrant protein folding [61]. In a hospital-based case-control study in Italy, higher garlic consumption was strongly inversely associated with CRC risk, suggesting that dietary garlic intake may exert chemoprotective effects in humans through its phytochemical compounds [62].

### 2.3. Terpenoids

Certain medications, such as 5-FU, are widely used in the treatment of CRC despite their association with side effects, including cardiotoxicity, hepatotoxicity, and nephrotoxicity. These toxic effects may be mediated, at least in part, by oxidative alterations [63, 64]. To mitigate these negative impacts, research has explored the potential of dietary terpenoids. For instance, one study investigated the co-exposure of 5-FU with varying doses of lycopene on the Caco2 cell line. This combination demonstrated improved antioxidant parameters, as shown by increased catalase and glutathione levels, potentially enabling the cells to manage 5-FU-induced oxidative stress. Additionally, the study observed an upregulation of IFN-γ expression in the presence of both 5-FU and lycopene, suggesting a potential enhancement of the drug's antitumor effects [65].

### 2.4. Alkaloids

Despite limitations regarding solubility and bioavailability, certain alkaloids within this chemical class have exhibited promising efficacy in both experimental and clinical settings. Compounds such as vincristine, vinblastine, paclitaxel, and berberine exemplify this potential by targeting diverse cellular and molecular pathways that are aberrantly regulated in colon cancer cells (e.g., cell cycle) [14, 66, 67]. Theobromine is the main alkaloid of the cacao bean and has been studied in *in vitro* and *in vivo* settings. Shojaei-Zarghani et al. [68] demonstrated that theobromine effectively suppressed tumor growth in a rat model of colon cancer. Moreover, this alkaloid reduced the expression of genes related to signaling pathways altered in a malignant state, such as the Akt/GSK3β/β-catenin cascade. Notably, immunohistochemical analysis revealed a marked upregulation of the tumor suppressor gene *Apc* in rats treated with theobromine compared to control animals.

Based on the available preclinical and clinical evidence, dietary phytochemicals have shown direct effects on some of the features of CRC. Further randomized controlled studies are needed to determine effective and safe doses for different populations worldwide. Also, it is important to address CRC heterogeneity among populations to target effective and safe diet-based strategies [29].

## 3. CRC and Gut Microbiota

In the case of CRC, the relevant microbial communities reside in the intestinal mucosa [69], and they are collectively referred to as the “gut microbiota” or “gut microbiome.” This includes a diverse array of microorganisms, encompassing bacteria, archaea, fungi, bacteriophages, human viruses, and protozoa. Most of them play a critical role in overall human health, as they are responsible for maintaining immune function, digesting fiber, and synthesizing of some B and K vitamins, short-chain fatty acids (SCFAs) (propionate, butyrate, and acetate), and other metabolites [70, 71]. Disruptions in the structure and composition of gut microbiota (dysbiosis) are associated with intestinal dysfunctions such as colon inflammation and a leaky gut [72]. These effects create a local noxious environment with short- and long-term local or systemic consequences, predisposing individuals to a wide spectrum of conditions [71]. According to various studies, the dysregulation of the gut microbiota and its metabolites is involved in the onset and progression of CRC [72–74]. Environmental factors such as sleep quality, drug intake, physical activity, and diet are critical to ensure an appropriate host–microbiota relationship [75]. The complexity of this interaction extends to potential genetic predispositions favoring specific microbial compositions within the host [76].

Colon inflammation has been proposed as a critical player in the promotion of CRC, and certain specific dysbiotic bacteria boost this procarcinogenic environment [77]. Several studies have identified specific proinflammatory bacterial taxa associated with CRC development, including *Fusobacterium nucleatum* [78–80], *Bacteroides fragilis* [72], *Enterococcus faecalis*, *Escherichia coli*, *Streptococcus gallolyticus* [74], *Campylobacter jejuni*, *Peptostreptococcus anaerobius* [79], *Parvimonas micra* [78, 79, 81], *Akkermansia muciniphila* [78, 82], and *Collinsella tanakaei* [78]. Mechanistically, some of these microbial species produce a repertoire of metabolites (e.g., Fap2, colibactin, and polyamines) that promote immune dysfunction at the colonic level [74, 83]. A study carried out by Coker et al. [79] identified distinct signatures of microbiota-derived metabolites associated with the transition to a neoplastic state in CRC. The metabolomic analysis revealed significant increases in norvaline, myristic acid, and proteinogenic amino acids in CRC patients compared to controls, while butyric acid levels showed a noteworthy decline. Therefore, the specific signatures of altered gut microbiota [84] and microbiota-derived metabolites could be harnessed for early diagnosis [85] and personalized dietary interventions [86].

An inverse association exists between the enrichment and abundance of specific beneficial bacteria and the development of CRC. Particularly, *Streptococcus thermophilus* [87], *Lactobacillus rhamnosus* GG [88], *Lactobacillus plantarum* L168 [89], and *Lactobacillus gallinarum* [90] have shown anti-CRC activities. Also, although contradictory with the findings mentioned in the previous paragraph, some studies have reported reduced levels of *Akkermansia muciniphila* in patients with CRC [76, 91].

One plausible mechanistic explanation that justifies their beneficial presence is their ability to metabolize dietary fiber and other indigestible food components, producing bioactive metabolites that mitigate inflammation. Prominent examples include the SCFAs butyrate, propionate, and acetate [92] and the indole-3-lactic acid (I3LA) [89]. Butyrate, in particular, has been shown to directly inhibit colonic tumorigenesis [93]. Another important point in the prevention of colon tumorigenesis is the appropriate maintenance of the colonic mucus layer. *Eubacterium coprostanoligenes* has showed positive effects in maintaining gut integrity in fiber-deprived animal models [69]. They could potentially be used as probiotics or as biomarkers in the fight against CRC.

Research on beneficial bacteria has translated into significant advancements in the prevention and treatment of CRC, leading to the rise of the term “probiotics.” In this context, Sugimura et al. [90] demonstrated that oral administration of *Lactobacillus gallinarum* exerts inhibitory effects on CRC in both *in vivo* and *in vitro* models. I3LA was identified as a key contributor to this antitumorigenic effect. Additionally, their findings suggest that *L. gallinarum* may promote the growth of other beneficial bacteria such as *L. helveticus* and *L. reuteri*, potentially suppressing pathogenic bacterial populations. Additionally, a separate study reported that a chemically induced mouse model supplemented with *Lactiplantibacillus plantarum*-12 exhibited a significant reduction in inflammatory markers and colon cancer symptoms, alongside a shift toward a beneficial gut microbiota composition [94]. These findings collectively highlight the promising potential of utilizing specific probiotic bacteria for CRC management.

## 4. Interplay Between Phytochemicals and Gut Microbiota in CRC

**Figure 2:**
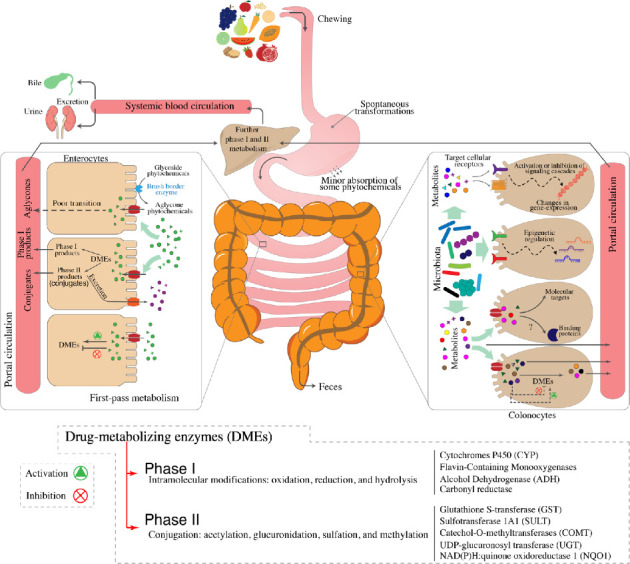
Metabolism of phytochemicals by the digestive system and gut microbiota. Phytochemicals undergo extensive chemical transformations at various levels within the gastrointestinal tract. Key contributors to this biotransformation process include the liver, small intestine, and colon, which harbor phase I and phase II drug-metabolizing enzymes (DMEs). These enzymes modulate the biological activity of dietary phytochemicals, either enhancing or diminishing their therapeutic potential. Additionally, the gut microbiota plays a crucial role in this metabolic process, producing metabolites with significant chemopreventive potential against CRC.

The low bioavailability of phytochemicals is attributed to their structural complexity, high molecular weight, limited solubility, and instability. Only 5%–10% of the ingested phytochemicals are absorbed by enterocytes, where they may undergo biotransformation via phase I and phase II metabolism. The remaining phytochemicals are transported to the colon, where they are also subjected to phase I and II metabolic enzymes and have the potential to modulate gut microbial communities (Figure 2). This modulation occurs because certain members of the microbiota metabolize phytochemicals into simpler compounds that, in addition to being absorbed by colonic epithelial cells, provide benefits to other commensal bacteria [15, 70]. Colonic microbial enzymes perform various chemical reactions on phytochemicals, including decarboxylation, deglycosylation, demethylation, dehydroxylation, ester cleavage, isomerization, and ring cleavage [95, 96]. These chemical modifications make phytochemicals attractive candidates for CRC chemoprevention, as some phytochemical-derived metabolites may target biochemical pathways involved in cancer progression [96, 97]. However, interindividual differences in phytochemical metabolism, influenced by variations in microbial communities, persist even among individuals exposed to the same diet [70, 98]. While these variations present challenges for the use of phytochemicals in CRC prevention and treatment, they also offer opportunities in the context of personalized onco-nutrition. Next, we will discuss the colonic and microbial metabolism of key members from the phytochemical classes outlined in Figure 1.

### 4.1. Phenolic Compounds

Phenolic compounds are present in a variety of foods, including raspberries, pomegranates, grapes, legumes, cereals, and green leafy vegetables [17]. These compounds are metabolized by drug-conjugating enzymes involved in phase II metabolism, and they can either inhibit or enhance the activity of these enzymes [99, 100]. For instance, the prenylation of flavonoids selectively enhances their cytotoxicity against colon cancer cells and influences drug-metabolizing enzymes, either directly or by modulating signaling cascades, such as the nuclear factor NF-κB and Keap1/Nrf2/ARE pathways [100].

Ellagitannins and ellagic acid are metabolized in the colon to produce urolithins A, B, C, and D, with urolithin A (Uro-A) being the most bioactive. Uro-A can induce shifts in energy metabolism, effectively counteracting the Warburg effect, which is typically observed in cancer cells. These effects are mediated through p53 signaling pathways [101]. *In vitro* studies have demonstrated that Uro-A has the potential to induce autophagy and inhibit metastasis in CRC cells [102]. Additionally, Giménez-Bastida et al. [97] showed that after long-term exposure, Uro-A selectively promotes cellular senescence in HCT-116 cells, rather than causing reversible cell cycle arrest or apoptosis. The authors suggested that this antitumor activity may serve as a chemopreventive strategy, potentially requiring lower doses through chronic consumption of ET-rich foods. Nonetheless, phase II metabolism of urolithins reduces their effectiveness against CRC cells [103], highlighting that the potential health benefits of these phytochemicals may be largely confined to the intestinal tract and their therapeutic effects may be dependent on both host and microbial metabolism.

Quercetin, a prominent and widely distributed flavonoid, is primarily metabolized via phase II pathways, resulting in the formation of conjugates including glucuronides, sulfates, and methylated derivatives [104]. Quercetin can also be found naturally in a conjugated form (e.g., quercetin-3-O-glucoside, quercetin-3-O-rutinoside, quercetin-4′-O-glucoside, and quercetin-3-O-glucoside-4′-O-glucoside). Cattivelli et al. [105] employed an interesting approach by testing the antiproliferative activity of parental quercetins, their colonic metabolites (low-molecular-weight phenolic acids), and a mixture of parental compounds and metabolites on colon cancer cell lines at physiological concentrations (100 μmol/L). Their results demonstrated variability in the effects, which were dependent on the type of compound, exposure time, and cell type, thereby underscoring the potential variability among individuals based on their metabotype (i.e., rapid or slow metabolism of parental compounds).

Foods such as soybeans contain the isoflavonoid daidzein, which is exclusively metabolized by gut microbiota to form equol, a highly bioactive metabolite with antioxidant and anti-CRC properties [98]. A study conducted by Polimeno et al. [20] demonstrated that daidzein metabolism differs in patients with sporadic colorectal adenomas compared to a control group (without proliferative lesions). This variation may be attributed to the presence of members of the *Bacteroides* genus. In addition, it has been suggested that members of the class *Coriobacteriia* such as *Hugonella massiliensis* and *Senegalimassilia faecalis* are capable of metabolizing daidzein [106]. Iino et al. [107] reported that *Asaccharobacter celatus* and *Slackia isoflavoniconvertens* are present in the gut microbiota of both equol producers and nonproducers. The key difference between these groups lies in the relative abundance of these bacterial species, a variation that may be attributed to the consumption of daidzein.

Another isoflavonoid present in soybeans is genistein, which modulates the gut microbiota and promotes the production of SCFAs, such as acetate, propionate, and butyrate [52]. They target different signaling cascades altered in CRC. Some studies tested their anti-CRC properties in combination with other phytochemicals. For instance, an *in vitro* study showed a synergetic anticancer effect of alkylresorcinol C21 in combination with butyrate, through the modulation of key molecular targets that induce apoptosis and autophagy [108].

Other legumes, including navy beans, contain phenolic compounds that modulate gut bacteria and their metabolites in individuals with a prior history of CRC [109]. A randomized controlled trial in overweight and obese CRC survivors found changes in the stool, urine, and plasma metabolome of patients who consumed a navy bean–containing diet (35 g/day in powder form) for 28 days. The metabolites were related to several biochemical pathways related to cancer prevention. The response was acute; whether this persists over time or not needs more research [110, 111]. Unlike fruits, legumes require cooking, a process that can alter or enhance the bioavailability of their phenolic compounds. Furthermore, it is important to consider which metabolites are generated by the microbiota when they are consumed either alone or in combination with other foods.

Fruits, including black raspberries, are rich sources of phenolic compounds, notably anthocyanins, which exhibit potential benefits for gut microbiota in the context of CRC [112]. A study in mice demonstrated that black raspberry exerts anti-CRC effects through modulation of gut microbiota composition and epigenetic demethylation of the *Sfrp2* gene, a negative regulator of Wnt signaling, thereby restoring its tumor-suppressive function [113]. Another investigation by Rodriguez et al. [114] revealed that supplementation with freeze-dried black raspberry significantly reduced colon tumorigenesis and modified fecal microbiota composition in a mouse model throughout tumor development. Notably, the shift toward a more beneficial microbiota composition was especially pronounced in animals maintained on a healthy diet. These studies suggest that anthocyanins might play a significant role in these beneficial effects.

Innovations within the probiotics industry require the incorporation of substances that ensure successful colonization and beneficial impacts of probiotics in the human gut. Phytochemicals may enhance the beneficial effects of probiotic bacteria. Compounds such as curcumin have emerged as promising candidates, as evidenced by their effects on the modulation of healthy gut microbes, including *Bifidobacterium* and *Lactobacillus* [115]. Curcumin-treated *Lactobacillus plantarum* has showed to significantly promote apoptosis in colon cancer cells *in vitro* compared to untreated bacteria. This augmented effect is attributed to curcumin's induction of anticancer metabolite release from the probiotic bacteria [116]. Further longitudinal studies across diverse human populations are essential to advance such innovations.

### 4.2. OSCs

OSCs present in vegetables, particularly cruciferous ones, are primarily biologically inactive in their intact form as glucosinolates, and they require hydrolysis by myrosinase, a β-thioglucoside glucohydrolase, to release their bioactive forms, such as ITCs and indoles. This hydrolysis can occur either in the food itself or via the action of myrosinase provided by the gut microbiota [117]. In the case of cooked foods, the role of the microbiota becomes particularly important, as heat inactivates the myrosinase enzyme [118]. For instance, glucobrassicins reaching the colon can be metabolized by bacteria such as *Enterococcus casseliflavus* CP1, leading to the generation of indole-3-carbinol [119]. This compound has demonstrated cytotoxic and proapoptotic effects *in vitro* using multiple CRC cell lines [120, 121]. Additionally, other studies have demonstrated that members of *Lactobacillus*, *Bifidobacterium*, and *Bacteroides* can also activate dietary glucosinolates to produce bioactive metabolites [118, 122].

It is currently recognized that OSCs may modulate the gut microbiome in a favorable manner. One study employed an *ex vivo* fecal incubation model using *in vitro*-digested broccoli sprouts to investigate the role of the gut microbiota in the production of bioactive compounds, such as nitrile sulforaphane and nitrile iberin. The analysis revealed significant variability in nitrile production and identified two distinct subpopulations of microbial communities among the fecal cultures, which were associated with differing nitrile concentrations. The highest levels of nitriles were linked to the *Clostridiaceae* family, while the lowest concentrations were observed in microbiota enriched with the *Enterobacteriaceae* family. These findings suggest that nitrile production may be influenced by the composition of the intestinal microbiota [123].

In another study, the gut microbiota and human digestive metabolome were characterized following the consumption of broccoli sprouts and Brussels sprouts, which are primary sources of OSCs. Significant changes were observed in several potentially beneficial bacterial families, including *Ruminococcaceae*, *Clostridiaceae*, *Lachnospiraceae*, *Erysipelotrichaceae*, *Eggerthellaceae*, *Peptostreptococcaceae*, *Streptococcaceae*, and *Enterococcaceae*. Additionally, the metabolomic analysis revealed enrichment in several bioactive metabolites, some of which exhibit variability between individuals [124]. These findings highlight the importance of multiomic approaches in analyzing dietary interventions on the microbiota and its metabolites.

### 4.3. Other Phytochemicals

In an experimental model, the phytochemical inositol hexaphosphate (IP6) prevented colon cancer metastasis by promoting changes in intestinal microbiota composition. The study showed that IP6 treatment restored the diversity of *Lactococcus lactis* and *Lactobacillus helveticus*, which may mediate these anticancer effects. Notably, IP6 is found in high amounts in grains and legumes, wheat bran, and flaxseeds [125].

## 5. Future Directions and Considerations

**Figure 3:**
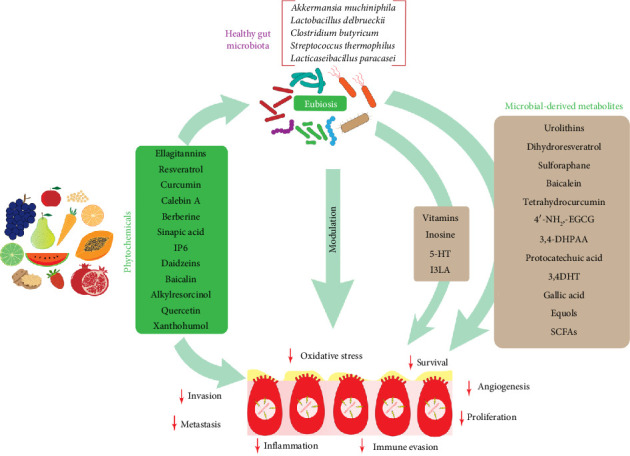
The interplay between phytochemicals and gut microbiota in CRC. Phytochemicals exert a direct influence on malignant events in cancer, while also facilitating the growth of a healthy microbiota, which in turn ameliorates the development of this malignancy directly and/or through the release of beneficial metabolites.

Approximately 5000 phytochemicals of nutritional significance have been identified [95], highlighting the necessity for rigorous, well-controlled human studies to address current knowledge gaps. Specific phytochemicals exert direct influence on CRC by targeting diverse biochemical pathways. Moreover, when these phytochemicals are metabolized by microbiota, they are transformed into bioactive anticancer substances (Figure 3). This mechanistic perspective aligns with the substantial body of epidemiological, experimental, and clinical evidence demonstrating that phytochemical-rich diets, such as the Mediterranean diet, are effective in preventing the onset, development, and invasiveness of CRC [10, 29].

The adoption of this chemopreventive strategy is feasible, as fruits, legumes, vegetables, nuts, and whole grains have been consumed in various healthy dietary interventions. Furthermore, the concentration of phytochemicals in these foods is low enough that it does not appear to pose harmful consequences. Additionally, foods containing phytochemicals are often rich in soluble fibers (e.g., prebiotics), which contribute to maintaining a healthy intestinal barrier. Indeed, the recommendations of the World Cancer Research Fund and the American Institute for Cancer Research include the consumption of fiber-rich foods, such as whole grains [126]. Dietitians should advocate for the incorporation of phytochemical-rich diets among CRC patients, grounded in scientific evidence [32]. Studies provide a mixed picture: a phase II study of Polyphenon E in high-risk colorectal subjects showed tolerability but did not show a statistically significant reduction in adenoma recurrence [44], and a large randomized trial of green tea extract over 3 years failed to achieve a clinically meaningful reduction in colorectal adenoma incidence (adenoma rate 55.7% placebo vs. 51.1% treated) [43]. On the other hand, recent preclinical work implicates microbiota–curcumin interactions: for example, Deng et al. [115] showed that curcumin's suppression of colorectal tumorigenesis is mediated in part by shifts in microbial composition and metabolite profiles. These findings highlight both the potential and the uncertainty of phytochemical use in nutrition and medicine, supporting the need for dose–response studies, reliable exposure biomarkers, and the stratification of participants by metabotype. As greater clarity emerges from such evidence, this dietary strategy could be more confidently incorporated into broader health promotion initiatives and supported by public health policies.

Currently, phytochemicals have not been classified as nutrients and therefore lack a recommended dietary intake (RDI). So, it becomes imperative to advocate for regulatory measures within this domain. Further research should establish optimal dosages of phytochemicals, considering factors such as age, sex, and physiological or pathological conditions, alongside the determination of maximum intake levels to prevent adverse effects arising from excessive consumption and potential toxicity due to cations and antinutrients present in plant-based diets. Strong analytical methods (e.g., UHPLC-MS/MS) for detecting inappropriate levels of metabolites and their altered derivatives are essential to determine what is truly safe and effective in humans [127]. Standardization of dietary recommendations for phytochemicals would also help move away from pseudoscientific claims that pose risks to individual well-being, particularly in countries with weak or absent regulation of the dietary supplement industry. We strongly emphasize the need for regulation to combat misinformation and prevent unintended side effects in CRC patients.

As we explore the quest for new and promising phytochemicals with anticancer properties, it is crucial to consider the flora diversity and wealth of traditional medicinal and gastronomical knowledge present in different countries around the world. Collaboration among nations with the necessary equipment, expertise, and know-how is imperative for the isolation/characterization/testing of phytochemicals and its microbial-derived metabolites. Additionally, the establishment of robust infrastructure to conduct clinical trials holds paramount importance in translating fundamental knowledge into tangible benefits for patients as in the case of functional foods such as prebiotics, probiotics, synbiotics, and postbiotics. Such efforts hold significance not only for CRC but also for other pathologies characterized by an inflammatory component, thereby amplifying the potential impact of phytochemical-based interventions.

We strongly recommend conducting specific population-based studies to comprehensively understand the complex dynamics of microbiota in the context of phytochemical intake and CRC. Additionally, the application of genetic engineering tools holds promise for the identification of exclusive enzymes derived from phytochemical-metabolizing gut microbiota. The integration of omics technologies (e.g., nontargeted metabolomics) would significantly enhance our understanding of patient-specific interventions [110, 111] and facilitate the metabotyping of individuals for the development of effective diet-based anticancer strategies [128].

It is crucial to recognize that although phytochemicals demonstrate potential in the prevention and treatment of various cancers, their effects cannot be solely attributed to individual compounds. Instead, these effects are likely the result of synergistic interactions among the phytochemicals themselves [129], other dietary components or metabolites [108], and anticancer drugs [101]. Polyherbal combinations, even when not derived from edible plant parts, may contribute to boosting anticancer immunity. Although still in the early stages of investigation, this approach offers valuable insights into the search for novel synergistic action [130]. Moreover, it is crucial to recognize that in the context of cancer, the efficacy of phytochemicals is influenced by factors such as current treatment strategies and other lifestyle factors. Therefore, a comprehensive approach that considers the multifactorial aspects of CRC is necessary to combat this devastating condition.

## Data Availability

Data sharing is not applicable to this article as no datasets were generated or analyzed during the current study.

## References

[1] International Agency for Research on Cancer (2024). Global Cancer Observatory: Cancer Today. https://gco.iarc.who.int/today/.

[2] Siegel R. L., Miller K. D., Wagle N. S., Jemal A. (2023). Cancer Statistics, 2023. *CA: A Cancer Journal for Clinicians*.

[3] American Cancer Society (2023). *Colorectal Cancer Facts & Figures 2023–2025*.

[4] De S., Paul S., Manna A. (2023). Phenolic Phytochemicals for Prevention and Treatment of Colorectal Cancer: A Critical Evaluation of *In Vivo* Studies. *Cancers*.

[5] Gavrilas L. I., Cruceriu D., Mocan A., Loghin F., Miere D., Balacescu O. (2022). Plant-Derived Bioactive Compounds in Colorectal Cancer: Insights From Combined Regimens With Conventional Chemotherapy to Overcome Drug-Resistance. *Biomedicines*.

[6] Keum N., Giovannucci E. (2019). Global Burden of Colorectal Cancer: Emerging Trends, Risk Factors and Prevention Strategies. *Nature Reviews Gastroenterology & Hepatology*.

[7] Yang T., Li X., Montazeri Z. (2019). Gene-Environment Interactions and Colorectal Cancer Risk: An Umbrella Review of Systematic Reviews and Meta-Analyses of Observational Studies. *International Journal of Cancer*.

[8] Mármol I., Sánchez-de-Diego C., Pradilla Dieste A., Cerrada E., Rodriguez Yoldi M. J. (2017). Colorectal Carcinoma: A General Overview and Future Perspectives in Colorectal Cancer. *International Journal of Molecular Sciences*.

[9] Hossain M. S., Karuniawati H., Jairoun A. A. (2022). Colorectal Cancer: A Review of Carcinogenesis, Global Epidemiology, Current Challenges, Risk Factors, Preventive and Treatment Strategies. *Cancers*.

[10] McLeod A., Wolf P., Chapkin R. S. (2023). Design of the Building Research in CRC Prevention (BRIDGE-CRC) Trial: A 6 Month, Parallel Group Mediterranean Diet and Weight Loss Randomized Controlled Lifestyle Intervention Targeting the Bile Acid-Gut Microbiome Axis to Reduce Colorectal Cancer Risk Among African American/Black Adults With Obesity. *Trials*.

[11] Mahmod A. I., Haif S. K., Kamal A., Al-ataby I. A., Talib W. H. (2022). Chemoprevention Effect of the Mediterranean Diet on Colorectal Cancer: Current Studies and Future Prospects. *Frontiers in Nutrition*.

[12] Thakur M., Singh K., Khedkar R., Prakash B. (2020). Phytochemicals: Extraction Process, Safety Assessment, Toxicological Evaluations, and Regulatory Issues. *Functional and Preservative Properties of Phytochemicals*.

[13] Taştemur Ş., Hacısüleyman L., Karataş Ö., Yulak F., Ataseven H. (2023). Anticancer Activity of Sinapic Acid by Inducing Apoptosis in HT-29 Human Colon Cancer Cell Line. *Canadian Journal of Physiology and Pharmacology*.

[14] Sun Q., Tao Q., Ming T. (2023). Berberine is a Suppressor of Hedgehog Signaling Cascade in Colorectal Cancer. *Phytomedicine*.

[15] Santhiravel S., Bekhit A. E. D. A., Mendis E. (2022). The Impact of Plant Phytochemicals on the Gut Microbiota of Humans for a Balanced Life. *International Journal of Molecular Sciences*.

[16] Kumar A., Nirmal P., Kumar M. (2023). Major Phytochemicals: Recent Advances in Health Benefits and Extraction Method. *Molecules*.

[17] Neveu V., Perez-Jimenez J., Vos F. (2010). Phenol-Explorer: An Online Comprehensive Database on Polyphenol Contents in Foods. *Database*.

[18] Afrin S., Giampieri F., Gasparrini M. (2020). Dietary Phytochemicals in Colorectal Cancer Prevention and Treatment: A Focus on the Molecular Mechanisms Involved. *Biotechnology Advances*.

[19] Zhan L., Su F., Li Q. (2023). Phytochemicals Targeting Glycolysis in Colorectal Cancer Therapy: Effects and Mechanisms of Action. *Frontiers in Pharmacology*.

[20] Polimeno L., Barone M., Mosca A. (2020). Soy Metabolism by Gut Microbiota From Patients With Precancerous Intestinal Lesions. *Microorganisms*.

[21] Yin X.-F., Ye T., Chen H.-L. (2024). The Microbiome Compositional and Functional Differences Between Rectal Mucosa and Feces. *Microbiology Spectrum*.

[22] Hanahan D., Weinberg R. A. (2011). Hallmarks of Cancer: The next Generation. *Cell*.

[23] Hanahan D. (2022). Hallmarks of Cancer: New Dimensions. *Cancer Discovery*.

[24] Hanahan D., Weinberg R. A. (2000). The Hallmarks of Cancer. *Cell*.

[25] George B. P., Chandran R., Abrahamse H. (2021). Role of Phytochemicals in Cancer Chemoprevention: Insights. *Antioxidants*.

[26] Delgado-Gonzalez P., Garza-Treviño E. N., de la Garza Kalife D. A., Quiroz Reyes A., Hernández-Tobías E. A. (2023). Bioactive Compounds of Dietary Origin and Their Influence on Colorectal Cancer as Chemoprevention. *Life (Basel)*.

[27] Surh Y. J. (2003). Cancer Chemoprevention With Dietary Phytochemicals. *Nature Reviews Cancer*.

[28] Hidaka A., Harrison T. A., Cao Y. (2020). Intake of Dietary Fruit, Vegetables, and Fiber and Risk of Colorectal Cancer According to Molecular Subtypes: A Pooled Analysis of 9 Studies. *Cancer Research*.

[29] Borgas P., Gonzalez G., Veselkov K., Mirnezami R. (2021). Phytochemically Rich Dietary Components and the Risk of Colorectal Cancer: A Systematic Review and Meta-Analysis of Observational Studies. *World Journal of Clinical Oncology*.

[30] Lee J., Shin A., Oh J. H., Kim J. (2017). Colors of Vegetables and Fruits and the Risks of Colorectal Cancer. *World Journal of Gastroenterology*.

[31] Wu Z. Y., Chen J. L., Li H., Su K., Han Y. W. (2023). Different Types of Fruit Intake and Colorectal Cancer Risk: A Meta-Analysis of Observational Studies. *World Journal of Gastroenterology*.

[32] Alzate-Yepes T., Pérez-Palacio L., Martínez E., Osorio M. (2023). Mechanisms of Action of Fruit and Vegetable Phytochemicals in Colorectal Cancer Prevention. *Molecules*.

[33] Ding S., Xu S., Fang J., Jiang H. (2020). The Protective Effect of Polyphenols for Colorectal Cancer. *Frontiers in Immunology*.

[34] Kim N., Kwon J., Shin U. S., Jung J. (2023). Stimulatory Anticancer Effect of Resveratrol Mediated by G Protein-Coupled Estrogen Receptor in Colorectal Cancer. *Biomolecules & Therapeutics (Seoul)*.

[35] Chen M., Tan A., Li J. (2023). Curcumin Represses Colorectal Cancer Cell Proliferation by Triggering Ferroptosis via PI3K/Akt/mTOR Signaling. *Nutrition and Cancer*.

[36] Chen X. X., Lam K. K. H., Feng Y. B. (2018). Ellagitannins from Pomegranate Ameliorates 5-Fluorouracil-Induced Intestinal Mucositis in Rats While Enhancing Its Chemotoxicity Against HT-29 Colorectal Cancer Cells Through Intrinsic Apoptosis Induction. *Journal of Agricultural and Food Chemistry*.

[37] do Carmo M. A. V., Fidelis M., de Oliveira P. F. (2021). Ellagitannins From Jabuticaba (*Myrciaria jaboticaba*) Seeds Attenuated Inflammation, Oxidative Stress, Aberrant Crypt Foci, and Modulated Gut Microbiota in Rats With 1,2 Dimethyl Hydrazine-Induced Colon Carcinogenesis. *Food and Chemical Toxicology*.

[38] Lv J. L., Tan Y. J., Ren Y. S. (2024). Procyanidin C1 Inhibits Tumor Growth and Metastasis in Colon Cancer via Modulating miR-501-3p/HIGD1A Axis. *Journal of Advanced Research*.

[39] Ganesan K., Jayachandran M., Xu B. (2020). Diet-Derived Phytochemicals Targeting Colon Cancer Stem Cells and Microbiota in Colorectal Cancer. *International Journal of Molecular Sciences*.

[40] Shuvalov O., Kirdeeva Y., Daks A. (2023). Phytochemicals Target Multiple Metabolic Pathways in Cancer. *Antioxidants*.

[41] Hu S. M., Yao X. H., Hao Y. H., Pan A. H., Zhou X. W. (2020). 8-Gingerol Regulates Colorectal Cancer Cell Proliferation and Migration Through the EGFR/STAT/ERK Pathway. *International Journal of Oncology*.

[42] Luo K. W., Xia J., Cheng B. H., Gao H. C., Fu L. W., Luo X. L. (2020). Tea Polyphenol EGCG Inhibited Colorectal-Cancer-Cell Proliferation and Migration via Downregulation of STAT3. *Gastroenterology Report*.

[43] Seufferlein T., Ettrich T. J., Menzler S. (2022). Green Tea Extract to Prevent Colorectal Adenomas, Results of a Randomized, Placebo-Controlled Clinical Trial. *American Journal of Gastroenterology*.

[44] Sinicrope F. A., Viggiano T. R., Buttar N. S. (2021). Randomized Phase II Trial of Polyphenon E Versus Placebo in Patients at High Risk of Recurrent Colonic Neoplasia. *Cancer Prevention Research*.

[45] Pajari A. M., Päivärinta E., Paavolainen L. (2016). Ellagitannin-Rich Cloudberry Inhibits Hepatocyte Growth Factor Induced Cell Migration and Phosphatidylinositol 3-Kinase/AKT Activation in Colon Carcinoma Cells and Tumors in Min Mice. *Oncotarget*.

[46] Nuñez-Sánchez M. A., González-Sarrías A., García-Villalba R. (2017). Gene Expression Changes in Colon Tissues From Colorectal Cancer Patients Following the Intake of an Ellagitannin-Containing Pomegranate Extract: A Randomized Clinical Trial. *The Journal of Nutritional Biochemistry*.

[47] Cao Q., Tian Y., Deng Z., Yang F., Chen E. (2024). Epigenetic Alteration in Colorectal Cancer: Potential Diagnostic and Prognostic Implications. *International Journal of Molecular Sciences*.

[48] Li J. S., Riggins K., Yang L. (2025). DNA Methylation Profiling at Base-Pair Resolution Reveals Unique Epigenetic Features of Early-Onset Colorectal Cancer in Underrepresented Populations. *Clinical Epigenetics*.

[49] Rodger E. J., Gimenez G., Ajithkumar P. (2023). An Epigenetic Signature of Advanced Colorectal Cancer Metastasis. *iScience*.

[50] Omar A., Govan D., Penny C. (2023). Epigenetic Regulation in Colorectal Cancer: The Susceptibility of microRNAs 145, 143 and 133b to DNA Demethylation and Histone Deacetylase Inhibitors. *PLoS One*.

[51] Hou Q., Huang J., Zhao L. (2023). Dietary Genistein Increases Microbiota-Derived Short Chain Fatty Acid Levels, Modulates Homeostasis of the Aging Gut, and Extends Healthspan and Lifespan. *Pharmacological Research*.

[52] Zhang Y., Li Q., Chen H. (2013). DNA Methylation and Histone Modifications of Wnt Genes by Genistein During Colon Cancer Development. *Carcinogenesis*.

[53] Zhu J., Ren J., Tang L. (2018). Genistein Inhibits Invasion and Migration of Colon Cancer Cells by Recovering WIF1 Expression. *Molecular Medicine Reports*.

[54] Zhao J., Li G., Ren Y. (2023). Ellagic Acid Inhibits Human Colon Cancer HCT-116 Cells by Regulating Long Noncoding RNAs. *Anti-Cancer Drugs*.

[55] Brockmueller A., Girisa S., Kunnumakkara A. B., Shakibaei M. (2023). Resveratrol Modulates Chemosensitisation to 5-FU via β1-Integrin/HIF-1α Axis in CRC Tumor Microenvironment. *International Journal of Molecular Sciences*.

[56] Brockmueller A., Samuel S. M., Mazurakova A., Büsselberg D., Kubatka P., Shakibaei M. (2023). Curcumin, Calebin A and Chemosensitization: How Are They Linked to Colorectal Cancer?. *Life Sciences*.

[57] Ağagündüz D., Şahin T. Ö., Yılmaz B., Ekenci K. D., Duyar Özer Ş., Capasso R. (2022). Cruciferous Vegetables and Their Bioactive Metabolites: From Prevention to Novel Therapies of Colorectal Cancer. *Evidence-Based Complementary and Alternative Medicine*.

[58] Cuellar-Nuñez M. L., Luzardo-Ocampo I., Lee-Martínez S. (2022). Isothiocyanate-Rich Extracts From Cauliflower (*Brassica oleracea* Var. Botrytis) and Radish (*Raphanus sativus*) Inhibited Metabolic Activity and Induced ROS in Selected Human HCT116 and HT-29 Colorectal Cancer Cells. *International Journal of Environmental Research and Public Health*.

[59] Kim J. S., Han S., Kim H. (2022). Anticancer Effects of High Glucosinolate Synthesis Lines of *Brassica rapa* on Colorectal Cancer Cells. *Antioxidants*.

[60] Moy K. A., Yuan J. M., Chung F. L. (2008). Urinary Total Isothiocyanates and Colorectal Cancer: A Prospective Study of Men in Shanghai, China. *Cancer Epidemiology, Biomarkers & Prevention*.

[61] Zhang Y., Liu X., Ruan J., Zhuang X., Zhang X., Li Z. (2020). Phytochemicals of Garlic: Promising Candidates for Cancer Therapy. *Biomedicine & Pharmacotherapy*.

[62] Speciani M. C., Gargari G., Penagini R. (2023). Garlic Consumption in Relation to Colorectal Cancer Risk and to Alterations of Blood Bacterial DNA. *European Journal of Nutrition*.

[63] da Silva M. C., Fabiano L. C., da Costa Salomão K. C. (2023). A Rodent Model of Human-Dose-Equivalent 5-Fluorouracil: Toxicity in the Liver, Kidneys, and Lungs. *Antioxidants*.

[64] Sara J. D., Kaur J., Khodadadi R. (2018). 5-Fluorouracil and Cardiotoxicity: A Review. *Therapeutic Advances in Medical Oncology*.

[65] Alhoshani N. M., Al-Zharani M., Almutairi B. (2022). Antioxidant and Anti-Inflammatory Activities of Lycopene Against 5-Fluorouracil-Induced Cytotoxicity in Caco2 Cells. *Saudi Pharmaceutical Journal*.

[66] Khan H., Alam W., Alsharif K. F., Aschner M., Pervez S., Saso L. (2022). Alkaloids and Colon Cancer: Molecular Mechanisms and Therapeutic Implications for Cell Cycle Arrest. *Molecules*.

[67] Olofinsan K., Abrahamse H., George B. P. (2023). Therapeutic Role of Alkaloids and Alkaloid Derivatives in Cancer Management. *Molecules*.

[68] Shojaei-Zarghani S., Rafraf M., Yari Khosroushahi A., Sheikh-Najafi S. (2020). Effectiveness of Theobromine on Inhibition of 1,2-Dimethylhydrazine-Induced Rat Colon Cancer by Suppression of the Akt/GSK3β/β-Catenin Signaling Pathway. *Journal of Functional Foods*.

[69] Bai D., Sun T., Zhao J. (2021). Oroxylin A Maintains the Colonic Mucus Barrier to Reduce Disease Susceptibility by Reconstituting a Dietary Fiber-Deprived Gut Microbiota. *Cancer Letters*.

[70] Kwon C., Ediriweera M. K., Kim Cho S. (2023). Interplay Between Phytochemicals and the Colonic Microbiota. *Nutrients*.

[71] Fan Y., Pedersen O. (2021). Gut Microbiota in Human Metabolic Health and Disease. *Nature Reviews Microbiology*.

[72] Spigaglia P., Barbanti F., Germinario E. A. P. (2023). Comparison of Microbiological Profile of Enterotoxigenic *Bacteroides fragilis* (ETBF) Isolates From Subjects With Colorectal Cancer (CRC) or Intestinal Pre-Cancerous Lesions Versus Healthy Individuals and Evaluation of Environmental Factors Involved in Intestinal Dysbiosis. *Anaerobe*.

[73] Busi S. B., Lei Z., Sumner L. W., Amos-Landgraf J. M. (2023). Integrated Multi-Omic Analyses Provide Insight Into Colon Adenoma Susceptibility Modulation by the Gut Microbiota. *mSystems*.

[74] Qu R., Zhang Y., Ma Y. (2023). Role of the Gut Microbiota and Its Metabolites in Tumorigenesis or Development of Colorectal Cancer. *Advanced Science*.

[75] Gacesa R., Kurilshikov A., Vich Vila A. (2022). Environmental Factors Shaping the Gut Microbiome in a Dutch Population. *Nature*.

[76] Colombo F., Illescas O., Noci S. (2022). Gut Microbiota Composition in Colorectal Cancer Patients is Genetically Regulated. *Scientific Reports*.

[77] Bernardazzi C., Castelo-Branco M. T. L., Pêgo B. (2022). The P2X7 Receptor Promotes Colorectal Inflammation and Tumorigenesis by Modulating Gut Microbiota and the Inflammasome. *International Journal of Molecular Sciences*.

[78] Chen H., Jiao J., Wei M. (2022). Metagenomic Analysis of the Interaction Between the Gut Microbiota and Colorectal Cancer: A Paired-Sample Study Based on the GMrepo Database. *Gut Pathogens*.

[79] Coker O. O., Liu C., Wu W. K. K. (2022). Altered Gut Metabolites and Microbiota Interactions Are Implicated in Colorectal Carcinogenesis and Can Be Non-Invasive Diagnostic Biomarkers. *Microbiome*.

[80] Chen S., Zhang L., Li M. (2022). *Fusobacterium nucleatum* Reduces METTL3-Mediated m6A Modification and Contributes to Colorectal Cancer Metastasis. *Nature Communications*.

[81] Zhao L., Zhang X., Zhou Y. (2022). *Parvimonas micra* Promotes Colorectal Tumorigenesis and is Associated With Prognosis of Colorectal Cancer Patients. *Oncogene*.

[82] Wang L., Tu Y. X., Chen L. (2023). Male-Biased Gut Microbiome and Metabolites Aggravate Colorectal Cancer Development. *Advanced Science*.

[83] Hanus M., Parada-Venegas D., Landskron G. (2021). Immune System, Microbiota, and Microbial Metabolites: The Unresolved Triad in Colorectal Cancer Microenvironment. *Frontiers in Immunology*.

[84] Avuthu N., Guda C. (2022). Meta-Analysis of Altered Gut Microbiota Reveals Microbial and Metabolic Biomarkers for Colorectal Cancer. *Microbiology Spectrum*.

[85] Zwezerijnen-Jiwa F. H., Sivov H., Paizs P., Zafeiropoulou K., Kinross J. (2023). A Systematic Review of Microbiome-Derived Biomarkers for Early Colorectal Cancer Detection. *Neoplasia*.

[86] Spivak I., Fluhr L., Elinav E. (2022). Local and Systemic Effects of Microbiome-Derived Metabolites. *EMBO Reports*.

[87] Li Q., Hu W., Liu W. X. (2021). *Streptococcus thermophilus* Inhibits Colorectal Tumorigenesis Through Secreting β-Galactosidase. *Gastroenterology*.

[88] Liotti F., Marotta M., Sorriento D. (2022). Probiotic *Lactobacillus rhamnosus* GG (LGG) Restrains the Angiogenic Potential of Colorectal Carcinoma Cells by Activating a Proresolving Program via Formyl Peptide Receptor 1. *Molecular Oncology*.

[89] Zhang Q., Zhao Q., Li T. (2023). *Lactobacillus plantarum*-Derived Indole-3-Lactic Acid Ameliorates Colorectal Tumorigenesis via Epigenetic Regulation of CD8+ T Cell Immunity. *Cell Metabolism*.

[90] Sugimura N., Li Q., Chu E. S. H. (2022). *Lactobacillus gallinarum* Modulates the Gut Microbiota and Produces Anti-Cancer Metabolites to Protect Against Colorectal Tumourigenesis. *Gut*.

[91] Gu Z. Y., Pei W. L., Zhang Y., Zhu J., Li L., Zhang Z. (2021). *Akkermansia muciniphila* in Inflammatory Bowel Disease and Colorectal Cancer. *Chinese Medical Journal*.

[92] Carretta M. D., Quiroga J., López R., Hidalgo M. A., Burgos R. A. (2021). Participation of Short-Chain Fatty Acids and Their Receptors in Gut Inflammation and Colon Cancer. *Frontiers in Physiology*.

[93] Kang J., Sun M., Chang Y. (2023). Butyrate Ameliorates Colorectal Cancer Through Regulating Intestinal Microecological Disorders. *Anti-Cancer Drugs*.

[94] Ma F., Sun M., Song Y. (2022). *Lactiplantibacillus plantarum*-12 Alleviates Inflammation and Colon Cancer Symptoms in AOM/DSS-Treated Mice Through Modulating the Intestinal Microbiome and Metabolome. *Nutrients*.

[95] Singh B., Mal G., Sharma D., Sharma R., Antony C. P., Kalra R. S. (2020). Gastrointestinal Biotransformation of Phytochemicals: Towards Futuristic Dietary Therapeutics and Functional Foods. *Trends in Food Science & Technology*.

[96] Feng X., Li Y., Brobbey Oppong M., Qiu F. (2018). Insights Into the Intestinal Bacterial Metabolism of Flavonoids and the Bioactivities of Their Microbe-Derived Ring Cleavage Metabolites. *Drug Metabolism Reviews*.

[97] Giménez-Bastida J. A., Ávila-Gálvez M. Á., Espín J. C., González-Sarrías A. (2020). The Gut Microbiota Metabolite Urolithin A, But Not Other Relevant Urolithins, Induces p53-Dependent Cellular Senescence in Human Colon Cancer Cells. *Food and Chemical Toxicology*.

[98] Lv J., Jin S., Zhang Y., Zhou Y., Li M., Feng N. (2024). Equol: A Metabolite of Gut Microbiota With Potential Antitumor Effects. *Gut Pathogens*.

[99] Lambert J. D., Sang S., Lu A. Y., Yang C. S. (2007). Metabolism of Dietary Polyphenols and Possible Interactions With Drugs. *Current Drug Metabolism*.

[100] Lněničková K., Šadibolová M., Matoušková P., Szotáková B., Skálová L., Boušová I. (2020). The Modulation of Phase II Drug-Metabolizing Enzymes in Proliferating and Differentiated CaCo-2 Cells by Hop-Derived Prenylflavonoids. *Nutrients*.

[101] Norden E., Heiss E. H. (2019). Urolithin A Gains in Antiproliferative Capacity by Reducing the Glycolytic Potential via the p53/TIGAR Axis in Colon Cancer Cells. *Carcinogenesis*.

[102] Zhao W., Shi F., Guo Z., Zhao J., Song X., Yang H. (2018). Metabolite of Ellagitannins, Urolithin A Induces Autophagy and Inhibits Metastasis in Human sw620 Colorectal Cancer Cells. *Molecular Carcinogenesis*.

[103] González-Sarrías A., Giménez-Bastida J. A., Núñez-Sánchez M. Á. (2014). Phase-II Metabolism Limits the Antiproliferative Activity of Urolithins in Human Colon Cancer Cells. *European Journal of Nutrition*.

[104] Chalet C., Hollebrands B., Duchateau G. S., Augustijns P. (2019). Intestinal Phase-II Metabolism of Quercetin in HT29 Cells, 3D Human Intestinal Tissues and in Healthy Volunteers: A Qualitative Comparison Using LC-IMS-MS and LC-HRMS. *Xenobiotica*.

[105] Cattivelli A., Conte A., Quercetins T. D. (2023). Chlorogenic Acids and Their Colon Metabolites Inhibit Colon Cancer Cell Proliferation at Physiologically Relevant Concentrations. *International Journal of Molecular Sciences*.

[106] Soukup S. T., Stoll D. A., Danylec N., Schoepf A., Kulling S. E., Huch M. (2021). Metabolism of Daidzein and Genistein by Gut Bacteria of the Class Coriobacteriia. *Foods*.

[107] Iino C., Shimoyama T., Iino K. (2019). Daidzein Intake Is Associated With Equol Producing Status Through an Increase in the Intestinal Bacteria Responsible for Equol Production. *Nutrients*.

[108] Zhao Y., Shi L., Hu C., Sang S. (2019). Wheat Bran for Colon Cancer Prevention: The Synergy Between Phytochemical Alkylresorcinol C21 and Intestinal Microbial Metabolite Butyrate. *Journal of Agricultural and Food Chemistry*.

[109] Sheflin A. M., Borresen E. C., Kirkwood J. S. (2017). Dietary Supplementation With Rice Bran or Navy Bean Alters Gut Bacterial Metabolism in Colorectal Cancer Survivors. *Molecular Nutrition & Food Research*.

[110] Baxter B. A., Oppel R. C., Ryan E. P. (2018). Navy Beans Impact the Stool Metabolome and Metabolic Pathways for Colon Health in Cancer Survivors. *Nutrients*.

[111] Zarei I., Baxter B. A., Oppel R. C., Borresen E. C., Brown R. J., Ryan E. P. (2021). Plasma and Urine Metabolite Profiles Impacted by Increased Dietary Navy Bean Intake in Colorectal Cancer Survivors: A Randomized-Controlled Trial. *Cancer Prevention Research*.

[112] Pan P., Lam V., Salzman N. (2017). Black Raspberries and Their Anthocyanin and Fiber Fractions Alter the Composition and Diversity of Gut Microbiota in F-344 Rats. *Nutrition and Cancer*.

[113] Chen L., Jiang B., Zhong C. (2018). Chemoprevention of Colorectal Cancer by Black Raspberry Anthocyanins Involved the Modulation of Gut Microbiota and SFRP2 Demethylation. *Carcinogenesis*.

[114] Rodriguez D. M., Hintze K. J., Rompato G. (2022). Dietary Supplementation With Black Raspberries Altered the Gut Microbiome Composition in a Mouse Model of Colitis-Associated Colorectal Cancer, Although With Differing Effects for a Healthy Versus a Western Basal Diet. *Nutrients*.

[115] Deng W., Xiong X., Lu M. (2024). Curcumin Suppresses Colorectal Tumorigenesis Through Restoring the Gut Microbiota and Metabolites. *BMC Cancer*.

[116] Gholipour F., Amini M., Baradaran B., Mokhtarzadeh A., Eskandani M. (2023). Anticancer Properties of Curcumin-Treated *Lactobacillus plantarum* Against the HT-29 Colorectal Adenocarcinoma Cells. *Scientific Reports*.

[117] Melim C., Lauro M. R., Pires I. M., Oliveira P. J., Cabral C. (2022). The Role of Glucosinolates From Cruciferous Vegetables (Brassicaceae) in Gastrointestinal Cancers: From Prevention to Therapeutics. *Pharmaceutics*.

[118] Sikorska-Zimny K., Beneduce L. (2021). The Metabolism of Glucosinolates by Gut Microbiota. *Nutrients*.

[119] Albaser A. A., Rossiter J. T., Luang-In V. (2023). Glucobrassicin Hydrolysis by Human Gut Bacteria and Putative Glycosyl Hydrolases Involved in the Process. *Food Agricultural Sciences and Technology*.

[120] Lee J., Lim H., Lee C., Park S. H., Nam M. (2021). Indole-3-Carbinol Inhibits the Proliferation of Colorectal Carcinoma LoVo Cells Through Activation of the Apoptotic Signaling Pathway. *Human & Experimental Toxicology*.

[121] Megna B. W., Carney P. R., Nukaya M., Geiger P., Kennedy G. D. (2016). Indole-3-Carbinol Induces Tumor Cell Death: Function Follows Form. *Journal of Surgical Research*.

[122] Liou C. S., Sirk S. J., Diaz C. A. C. (2020). A Metabolic Pathway for Activation of Dietary Glucosinolates by a Human Gut Symbiont. *Cell*.

[123] Bouranis J. A., Beaver L. M., Choi J. (2021). Composition of the Gut Microbiome Influences Production of Sulforaphane-Nitrile and Iberin-Nitrile From Glucosinolates in Broccoli Sprouts. *Nutrients*.

[124] Bouranis J. A., Beaver L. M., Jiang D. (2022). Interplay Between Cruciferous Vegetables and the Gut Microbiome: A Multi-Omic Approach. *Nutrients*.

[125] Lan T. T., Song Y., Liu X. H. (2022). IP6 Reduces Colorectal Cancer Metastasis by Mediating the Interaction of Gut Microbiota With Host Genes. *Frontiers in Nutrition*.

[126] World Cancer Research Fund/American Institute for Cancer Research (2018). Diet, Nutrition, Physical Activity and Colorectal Cancer. Continuous Update Project Expert Report. https://dietandcancerreport.org.

[127] Zahiruddin S., Wang Y. H., Kothapalli H. B., Avula B., Chittiboyina A. G., Khan I. A. (2025). An Ultrahigh-Performance Liquid Chromatography Coupled With Tandem Mass Spectrometry (UHPLC-MS/MS) Method for Toxicity and Safety Level Assessment of Oxygen Heterocyclic Compounds and Terpene in Cold-Pressed Grapefruit Essential Oils. *ACS Omega*.

[128] Hillesheim E., Brennan L. (2020). Metabotyping and Its Role in Nutrition Research. *Nutrition Research Reviews*.

[129] Rizeq B., Gupta I., Ilesanmi J., AlSafran M., Rahman M. M., Ouhtit A. (2020). The Power of Phytochemicals Combination in Cancer Chemoprevention. *Journal of Cancer*.

[130] Zahiruddin S., Parveen A., Khan W. (2022). Metabolomic Profiling and Immunomodulatory Activity of a Polyherbal Combination in Cyclophosphamide-Induced Immunosuppressed Mice. *Frontiers in Pharmacology*.

